# Complete Genome Sequences of Five Acinetobacter baumannii Phages from Abidjan, Côte d’Ivoire

**DOI:** 10.1128/MRA.01358-18

**Published:** 2019-01-03

**Authors:** Christiane Essoh, Jean-Philippe Vernadet, Gilles Vergnaud, Adama Coulibaly, Adèle Kakou-N’Douba, Assanvo S.-P. N’Guetta, Gregory Resch, Christine Pourcel

**Affiliations:** a Department of Biochemistry-Genetic, School of Biological Sciences, Université Peleforo Gon Coulibaly, Korhogo, Côte d’Ivoire; b Institute for Integrative Biology of the Cell (I2BC), CEA, CNRS, Université Paris-Sud, Université Paris-Saclay, Paris, France; c Laboratory of Bacteriology-Virology, Department of Microbiology, School of Medical Sciences, Université Félix Houphouët-Boigny, Abidjan, Côte d’Ivoire; d Laboratory of Genetic, School of Biosciences, Université Félix Houphouët-Boigny, Abidjan, Côte d’Ivoire; e Department of Fundamental Microbiology, University of Lausanne, Lausanne, Switzerland; University of Arizona

## Abstract

Five bacteriophages of Acinetobacter baumannii were isolated from sewage water in Abidjan, Côte d’Ivoire. Phages Aci01-1, Aci02-2, and Aci05 belong to an unclassified genus of the Myoviridae family, with double-stranded DNA (dsDNA) genomes, whereas Aci07 and Aci08 belong to the Fri1virus genus of the Podoviridae family of phages.

## ANNOUNCEMENT

**FIG 1:**
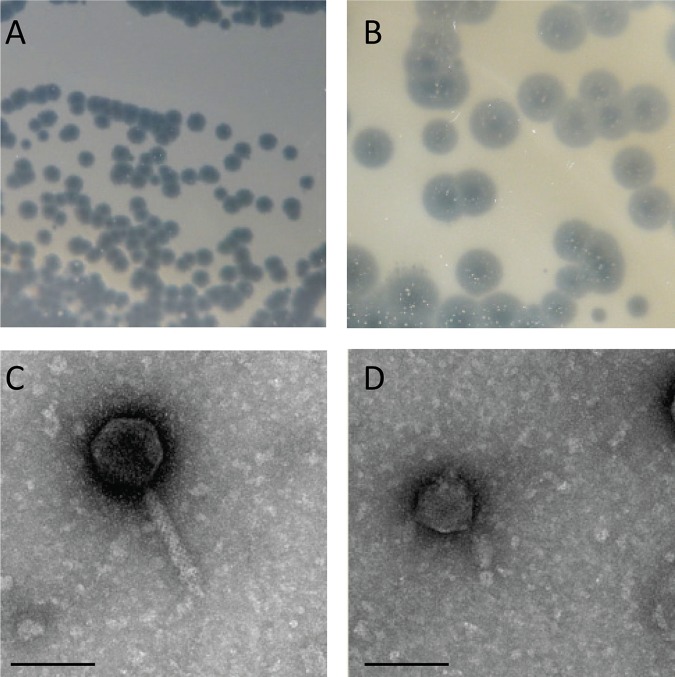
Plaque morphology of phage Aci05 (0.5 mm) (A) and phage Aci07 (2 mm) (B). EM of phage Aci05 (C) and phage Aci07 (D). Bar, 100 nm.

Using two clinical strains of Acinetobacter baumannii, B09 and Abidjan-46-62, we isolated five lytic bacteriophages from hospital sewage water in Abidjan, Côte d’Ivoire. Five milliliters of water was clarified by centrifugation at 2,500 × *g* for 20 min and then filtrated through a 0.45-µm filter. Two hundred microliters of sample was then incubated for 20 h in 2 ml LB medium with 20 µl of an overnight bacterial culture, and after centrifugation, the supernatant was spotted onto bacteria in soft LB agar. Phages recovered in lysis zones were amplified as previously described (1). Phages vB_AbaM_B09_Aci01-1 (short name, Aci01-1), vB_AbaM_B09_Aci02-2 (Aci02-2), and vB_AbaM_B09_Aci05 (Aci05) formed small clear plaques, whereas vB_AbaP_46-62_Aci07 (Aci07) and vB_AbaP_B09_Aci08 (Aci08) produced clear plaques with a large halo (Fig. 1A and B). The morphology of phage virions was determined using transmission electron microscopy (Fig. 1C and D). Phage suspensions were adsorbed on a grid coated with carbon (EMS, Hatfield, PA, USA) and then washed with distilled water, followed by staining with 1% uranyl acetate (Sigma, St. Louis, MO, USA) in H_2_O for 1 min. Electron micrographs (EM) were taken with a Philips CM100 transmission electron microscope (Thermo Fisher Scientific, Hillsboro, USA) at an acceleration voltage of 80 kV. EM for Aci01-1, Aci02-2, and Aci05 showed a 75 ± 4-nm head and a 140 ± 2-nm nonflexible contractile tail, which are characteristic of Myoviridae, and EM for Aci07 and Aci08 showed a 60 ± 2-nm icosahedral capsid with a short tail that is characteristic of Podoviridae. For genome purification, bacteriophages were lysed in lysis buffer (10 mM Tris [pH 7.8]; 10 mM EDTA; 10 mM NaCl; 0.5% SDS [wt/vol]) and treated with proteinase K at 50 µg ml^−1^ for 2 h at 50°C, followed by one phenol and one chloroform extraction and ethanol precipitation. Each phage genome was sequenced separately in an Illumina MiSeq 300-bp paired-end run with a 900-bp insert library, producing 2.7 to 5.5 million reads (800 to 1,700 Mb), including a variable proportion of reads derived from contaminating bacterial host DNA. Quality control was performed with FastQC version 0.11.5, and reads were trimmed with GeneiousR11 (Biomatters Ltd., Auckland, New Zealand) with default trim parameters. Assembly was performed on 10% of the reads using the Geneious assembler with the medium sensitivity/fast option to a single linear contig. The total amount of reads was then mapped to the contig to produce a high-quality sequence at a mean coverage of 1,000× or more. The presence of direct terminal repeats (DTRs) which induced a higher read coverage allowed for identification of the phage genome ends. Automatic annotation was performed with Rapid Annotations using Subsystems Technology (RAST) (2).

The Aci01-1, Aci02-2, and Aci05 genomes were 103,628 bp, 104,354 bp, and 102,789 bp long with DTRs of 1,184 bp, 1,198 bp, and 1,151 bp, respectively. A comparison was performed by aligning the genomes using MAFFT version 7 (3) as embedded in GeneiousR11. The average nucleotide identity (ANI) among the Aci01-1, Aci02-2, and Aci05 genomes was higher than 90% compared with less than 65% toward phage vB_AbaM_phiAbaA1 from Poland (GenBank accession number KJ628499) and phage Acibel004 from Belgium (GenBank accession number KJ473422) (4), two presently unclassified myoviruses (5). The genome size and architecture and the presence of a DTR and tRNAs suggest that these phages may be members of the Felixounavirinae subfamily (1, 6).

The Aci07 and Aci08 genomes were 42,330 bp and 42,067 bp long with 397-bp and 410-bp DTRs, respectively. As estimated by eye, the overall gene organization of Aci07 and Aci08 was that of ApiP_P1 (GenBank accession number MF033350) and AS12 (GenBank accession number KY268295), respectively, belonging to the genus Fris1virus of the Autographinivirinae subfamily (7). The ANI among these phages as determined by MAFFT alignment varied from 77% to 86%.

### Data availability.

The genome sequences of the five phages are available at GenBank under accession numbers MH800198 (Aci01-1), MH800199 (Aci02-2), MH746814 (Aci05), MH800200 (Aci07), and MH763831 (Aci08). The raw read archives have been deposited in the European Nucleotide Archive (ENA) under BioProject accession number PRJEB28456.
